# Investigation of Prognostic Markers of Lung Adenocarcinoma Based on Tumor Metabolism-Related Genes

**DOI:** 10.3389/fgene.2021.760506

**Published:** 2021-10-19

**Authors:** Chong Zhang, Zhehao He, Ling Cheng, Jinlin Cao

**Affiliations:** ^1^ Department of Thoracic Surgery, The First Affiliated Hospital, College of Medicine, Zhejiang University, Hangzhou, China; ^2^ Shanghai Engineering Research Center of Pharmaceutical Translation, Shanghai, China

**Keywords:** lung adenocarcinoma, prognosis prediction, GSEA enrichment analysis, TP53, immune infiltration

## Abstract

Lung adenocarcinoma (LUAD) is a prevalent cancer killer. Investigation on potential prognostic markers of LUAD is crucial for a patient’s postoperative planning. LUAD-associated datasets were acquired from Gene Expression Omnibus (GEO) as well as The Cancer Genome Atlas (TCGA). LUAD metabolism-associated differentially expressed genes were obtained, combining tumor metabolism-associated genes. COX regression analyses were conducted to build a five-gene prognostic model. Samples were divided into high- and low-risk groups by the established model. Survival analysis displayed favorable prognosis in the low-risk group in the training set. Favorable predictive performance of the model was discovered as hinted by receiver’s operative curve (ROC). Survival analysis and ROC analysis in the validation set held an agreement. Gene Set Enrichment Analysis (GSEA), tumor mutation bearing (TMB), and immune infiltration differential analysis were performed. The two groups displayed differences in glycolysis gluconeogenesis, P53 signaling pathway, etc. The high-risk group showed higher TP53 mutation frequency as well as TMB. The low-risk group displayed higher immune activity along with immune score. Altogether, this study casts light on further development of novel prognostic markers for LUAD.

## Introduction

Lung cancer (LC) is a leading cause of cancer-associated deaths and the commonest cancer worldwide (Chen et al., 2016). There is a lack of specific symptoms and tumor markers in the early stage of lung adenocarcinoma (LUAD). Most patients are in the late stage when diagnosed and develop lymph nodes and multiple metastases in other sites (Siegel et al., 2019). Major therapeutic methods for LUAD include surgical excision, platinum chemotherapy, radiotherapy, or/and targeted therapy. Unfortunately, LUAD patients have a poor prognosis, and terminal patients usually relapse in the early stage, with a 5 years overall survival (OS) lower than 20% (Torre et al., 2016; Siegel et al., 2021). Thus, the development of prognostic markers for LUAD is warranted.

Metabolism is a prerequisite for all life activities of an animated body, while tumor occurrence is often accompanied by reprogramming of cell metabolism. A tumor reprograms the metabolism pathway to meet the requirements for malignant cell biosynthesis and nutrition, which is regarded as one of the markers of cancers (DeBerardinis and Chandel, 2016; Pavlova and Thompson, 2016). Studies displayed two hallmarks of cancer metabolism: metabolic interactions with the microenvironment as well as alterations in metabolite-driven gene regulation (Pavlova and Thompson, 2016; Anastasiou, 2017). The following are typical examples: Enhanced glycolysis stimulates production of lactic acid, and the latter inhibits T cell proliferation in the tumor microenvironment (Fischer et al., 2007). Oscar et al. (Colegio et al., 2014) also found that massive lactic acid in the tumor microenvironment stimulates M2-like polarization of macrophages to accelerate cancer progression. Thus, further understanding of cancer metabolism pathway and finding key metabolism targets offer guidance for targeted therapy of cancer metabolism.

With the rapid development of biological technology and bioinformatics, the exploration of cancer diagnosis and prognostic biomarkers based on bioinformatics method has recently been in the limelight. Mo et al. (2020) identified and validated the prognosis potential of hypoxia-related feature genes in LUAD based on the hypoxia-related microenvironment. These genes may be new targets for immune therapy. Zhang et al. (2019) built a risk score model using 14 immune-related genes, presenting a rationale for the prognosis of diverse immunophenotypes. Gao et al. (2021) constructed a ferroptosis-associated gene signature using bioinformatics analysis and hinted at a possible option for LUAD treatment by targeting ferroptosis-associated genes. Therefore, it is promising to establish a prognostic model based on public data combining immunity, hypoxia, and other characteristics.

Here, a five-gene prognostic model was established based on mRNA expression data of LUAD in The Cancer Genome Atlas (TCGA)/Gene Expression Omnibus (GEO) using several bioinformatics methods. We also identified metabolism-associated prognostic markers in LUAD. This investigation offers a rationale for the development of prognostic biomarkers of LUAD.

## Materials and Methods

### Dataset Download and Processing

mRNA expression data (normal: 59, tumor: 535) in fragments per kilo-base of exon per million fragments mapped (FPKM) and count formats (normal: 59, tumor:535), clinical data, and single-nucleotide variant (SNV) data (VarScan2 Annotation, sample number: 561) were downloaded from TCGA (https://portal.gdc.cancer.gov/; October 20th, 2020). Dataset GSE72094 was accessed from GEO (https://www.ncbi.nlm.nih.gov/geo/) as the validation set. Raw data were provided by GPL15048 platform.

### Screening of Lung Adenocarcinoma Metabolism-Associated Genes and Gene Ontology and Kyoto Encyclopedia of Genes and Genomes Enrichment Analysis

Differential expression analysis was undertaken on the normal group and tumor group in the training set using “edgeR” package to screen differentially expressed genes (DEGs). The threshold value was set as |logFC| > 1.5 and false discovery rate (FDR) < 0.05 (Robinson et al., 2010). Tumor metabolism-associated gene sets compiled by Possemato et al. (2011) were downloaded from Pubmed (Supplementary Table S1). DEGs were intersected with tumor metabolism-associated genes to obtain DEGs associated with LUAD metabolism. Thereafter, Gene Ontology (GO) and Kyoto Encyclopedia of Genes and Genomes (KEGG) enrichment analyses were performed on metabolism-associated DEGs using “clusterprofiler” package (q value < 0.05) (Yu et al., 2012).

### Screening of Prognostic Feature Genes Associated With Metabolism in Lung Adenocarcinoma

Samples whose survival time is less than 30 days in TCGA-LUAD were removed. Univariate COX regression analysis was undertaken on metabolism-associated DEGs using “survival” package to obtain survival-related DEGs in LUAD (*p* < 0.05) (Modeling Survival Data, 2013). To avoid overfitting of the statistical model, “glmnet” package was used to perform LASSO COX regression analysis on the above-screened DEGs (Friedman et al., 2010). Penalty parameter “λ” was selected to remove genes with strong relevance through cross validation to reduce the complexity of the model. Finally, “survival” package was used to undertake multivariate COX regression analysis on the above genes. Prognostic feature genes associated with LUAD metabolism were identified. A risk score model was established, and the risk score was calculated by using the following formula:

$$Risk\;score=\overset{n}{\underset{i=1}{\sum }}exp_{i}\text{*}\beta_{i}$$ (1)

The number of prognostic feature genes associated with metabolism is denoted by *n*; the expression level of gene *i* is denoted by *exp*_*i*_; the regression coefficient of gene *i* is denoted by *β*_*i*_.

### Analysis of Predictive Performance of Risk Score

The risk scores of patients in TCGA-LUAD were calculated based on the expression levels of prognostic feature genes associated with metabolism. The patients were divided into high- and low-risk groups with median risk score as the threshold value. Survival curves of the two groups were drawn using “survival” package. Receiver’s operative curve (ROC) of patient’s 1-, 3-, and 5 years OS was drawn with “timeROC” package. The area under the curve (AUC) was calculated. The results were validated in the validation set to evaluate the predictive performance of the model (Blanche et al., 2013).

### Gene Set Enrichment Analysis on High- and Low-Risk Groups

Gene Set Enrichment Analysis (GSEA) enrichment analytics tool was accessed from http://www.gsea-msigdb.org/gsea/index.jsp. The signaling pathway enrichment in high- and low-risk groups was analyzed using GSEA software (*p* < 0.05) to differentiate biological functions in the two groups. The significance of the enrichment score was analyzed by permutation test (permutation test time: 1,000) (Subramanian et al., 2005).

### Tumor Mutation Bearing in Two Groups and Analysis of Mutation Genes in Lung Adenocarcinoma

Tumor mutation bearing (TMB) is defined as the total number of detected somatic cell gene coding errors, base substitutions, errors in gene insertion, or deletions per million bases (Yarchoan et al., 2017). The significance of TMB in the two groups in TCGA-LUAD was analyzed using Wilcoxon test. Mutation genes in the high- and low-risk groups were analyzed, combining SNV mutation data. Waterfall plots of the top 30 gene mutations in the two groups were drawn by R package “GenVisR” (Skidmore et al., 2016).

### Evaluation of Immune Infiltration in Two Groups

R package “estimate” was used to assess the stromal score, immune score, and tumor purity in LUAD samples in TCGA. Single simple GSEA (ssGSEA) analysis was performed on 29 immune cells using “GSVA” package to assess the immune infiltration levels of each tumor sample. Differential expression analysis was performed on immune infiltration levels in the two groups using Wilcoxon test (Barbie et al., 2009).

## Results

### Differentially Expressed Genes Identification and Enrichment Analyses

**FIGURE 1:**
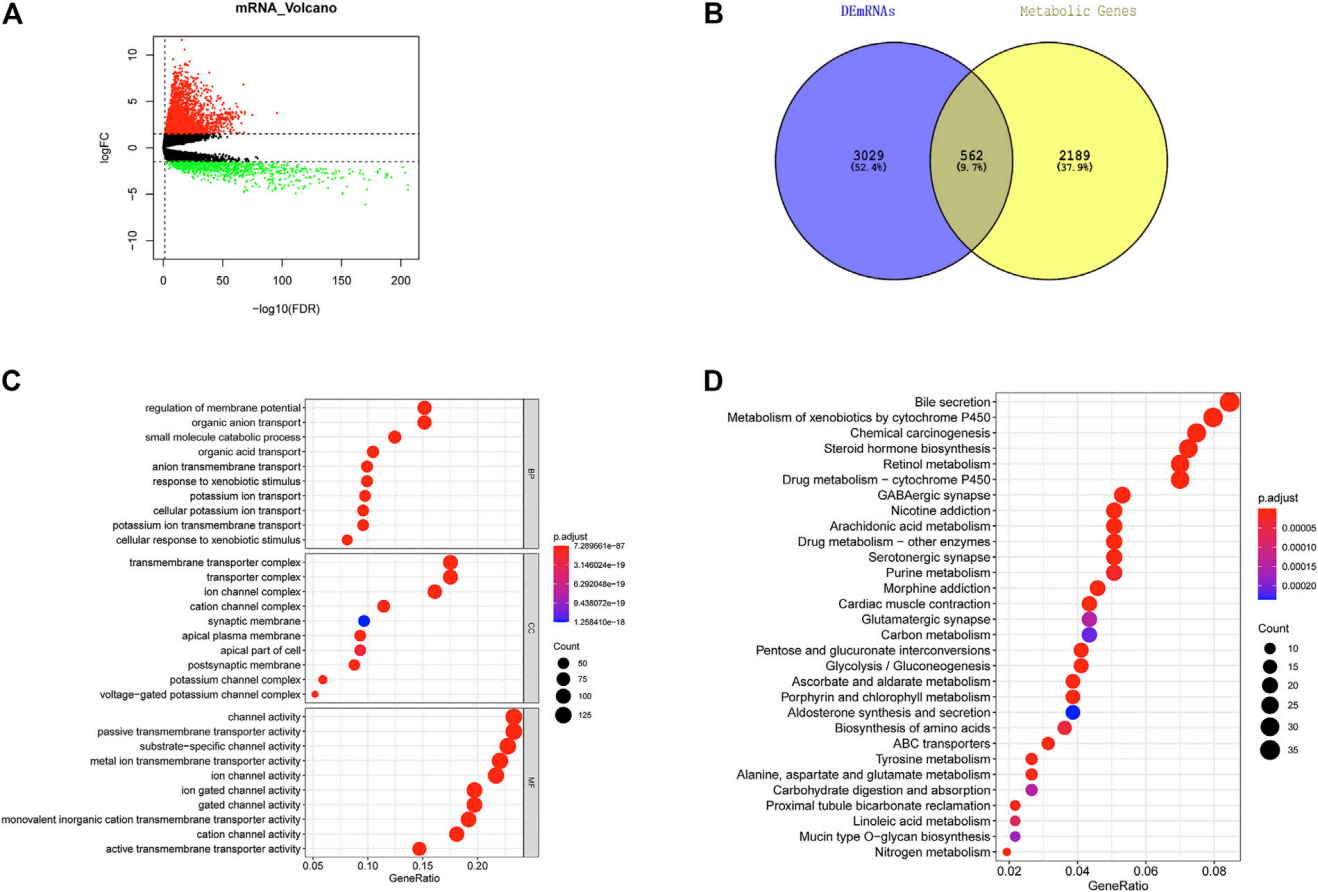
Screening of metabolism-associated DEGs in LUAD and functional enrichment analysis. **(A)** Volcano plot of differential expression analysis on tumor group and normal groups in TCGA-LUAD dataset. Red: significantly upregulated DEGs. Green: significantly downregulated DEGs. **(B)** Overlap of DEGs and metabolism-associated genes in LUAD to acquire metabolism-associated DEGs in LUAD. **(C)** Bubble diagram of GO enrichment analysis on DEGs associated with metabolism in LUAD. Nodes: enriched terms. The node size is proportional to the number of enriched genes; the deeper red color of node indicates the smaller *p* values. **(D)** Bubble diagram of KEGG enrichment analysis on DEGs associated with metabolism in LUAD. Nodes: enriched terms. The node size is proportional to the number of enriched genes; the deeper red color of node indicates the smaller *p* values.

Altogether, 3,591 DEGs were acquired through differential expression analysis on normal and tumor groups in TCGA-LUAD in the training set (|logFC| > 1.5, FDR <0.05), including 2,553 upregulated and 1,038 downregulated genes (Figure 1A). As shown in Figure 1B, 562 LUAD metabolism-associated DEGs were acquired by overlapping DEGs and tumor metabolism-associated gene sets. GO and KEGG enrichment analyses were undertaken on metabolism-associated DEGs in LUAD. GO enrichment analysis showed that these genes were mostly enriched in biological functions including regulation of membrane potential, small molecule catabolic process, organic acid transport, and cellular response to xenobiotic stimulus (Figure 1C). KEGG enrichment analysis showed that these genes were mostly enriched in signaling pathways including the metabolism of xenobiotics by cytochrome P450, retinol metabolism, drug metabolism-other enzymes, arachidonic acid metabolism, and purine metabolism (Figure 1D).

### Prognostic Model Construction Based on Feature Genes

**FIGURE 2:**
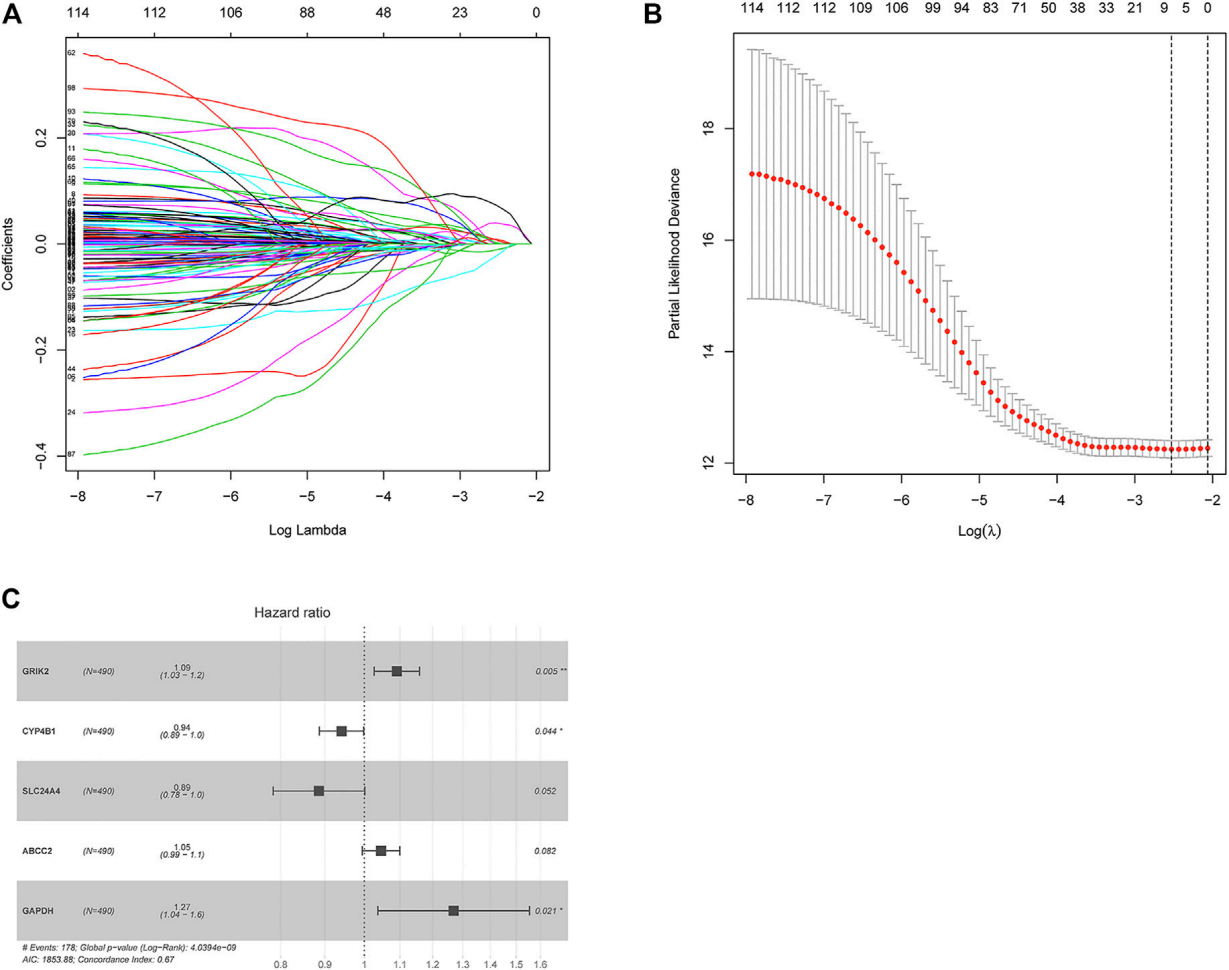
Construction of a five-gene based prognostic model. **(A)** The coefficients of 117 survival-related genes vary with the penalty parameter lambda in LASSO regression analysis. **(B)** Selection range of the optimal penalty parameter (λ) of LASSO COX regression model. The upper coordinate indicates the number of genes corresponding to different lambda values. **(C)** Forest plot of multivariate COX regression analysis. *p < 0.05. **p < 0.01.

Combining patient’s survival data in TCGA-LUAD in the training set, 562 DEGs associated with metabolism of LUAD were subjected to univariate COX regression analysis. Altogether, 117 genes relevant to survival were acquired (Supplementary Table S2). Optimal penalty parameter “λ” was chosen through cross validation. Eight metabolism-associated prognostic feature genes were acquired (Figures 2A,B). These eight feature genes were subjected to multivariate regression analysis. Lastly, five optimal prognostic feature genes associated with LUAD metabolism were obtained to establish a risk score model (Supplementary Table S3). Protective factors were CYP4B1 and SLC24A4. Hazard ratio (HR) was 0.94 and 0.89. Risk factors were CRIK2 (1.09), ABCC2 (1.05), and glyceraldehyde 3-phosphate dehydrogenase (GAPDH) (1.27) (Figure 2C).

### Evaluation of the Performance of the Five-Gene Based Prognostic Model

**FIGURE 3:**
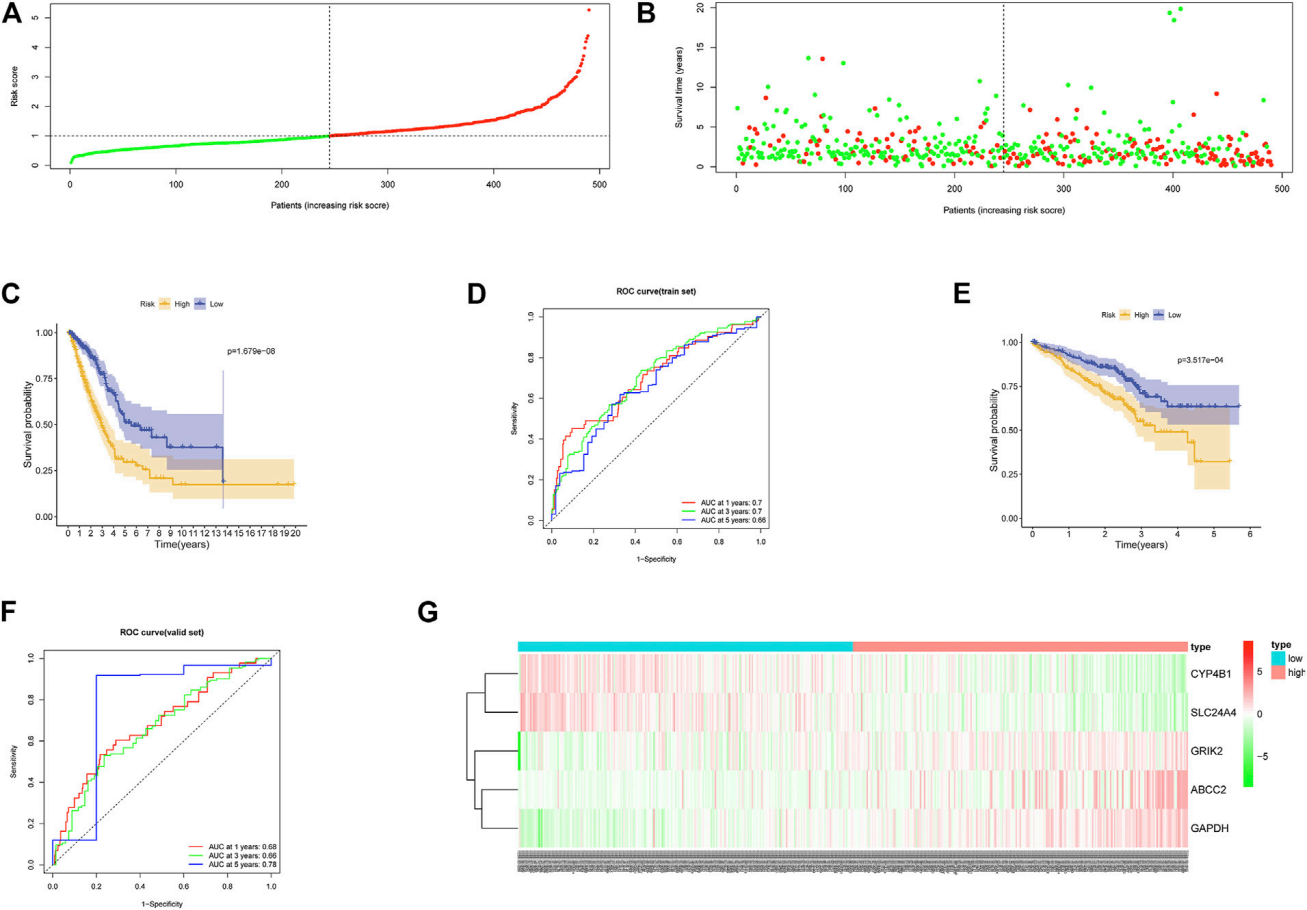
Performance of the prognostic model. **(A)** Distribution of risk score of each LUAD sample in the training set (green: patients having low-risk score; red: patients having high-risk score). **(B)** Scatter diagram of survival status of LUAD patients according to risk score (green: survived patients; red: dead patients). **(C)** Survival curves of high- and low-risk groups in the training set. **(D)** ROC curves of the prognostic model in the training set. **(E)** Survival curves of the high- and low-risk groups in the validation set. **(F)** ROC curves of the prognostic model in the validation set. **(G)** Heat map of the expression of the five feature genes in the high- and low-risk groups in the training set.

Risk scores of samples in TCGA-LUAD in the training set were calculated. Samples were then divided into high- and low-risk groups according to the median score. Meanwhile, we drew survival status plots, survival curves, and ROC curves of the two groups (Figures 3A–D). Survival analysis suggested poorer survival status in the high-risk group in comparison with the low-risk group. ROC curve showed that AUC values of 1-, 3-, and 5 years survival curves were 0.7, 0.7, and 0.66. The favorable prognosis predictive performance of the model was further proved by survival curve and ROC curve of GSE72094 in the validation set (Figures 3E,F). As shown by heat map of expression levels of five feature genes in the two groups, with the increasing of risk scores, the expression of risk factors (CRIK2, ABCC2, GAPDH) were gradually elevated, while the expression of protective factors (CYP4B1, SLC24A4) was decreased (Figure 3G). Overall, the constructed model could predict the LUAD patient’s prognosis well.

### Gene Set Enrichment Analysis Enrichment Analysis

**FIGURE 4:**
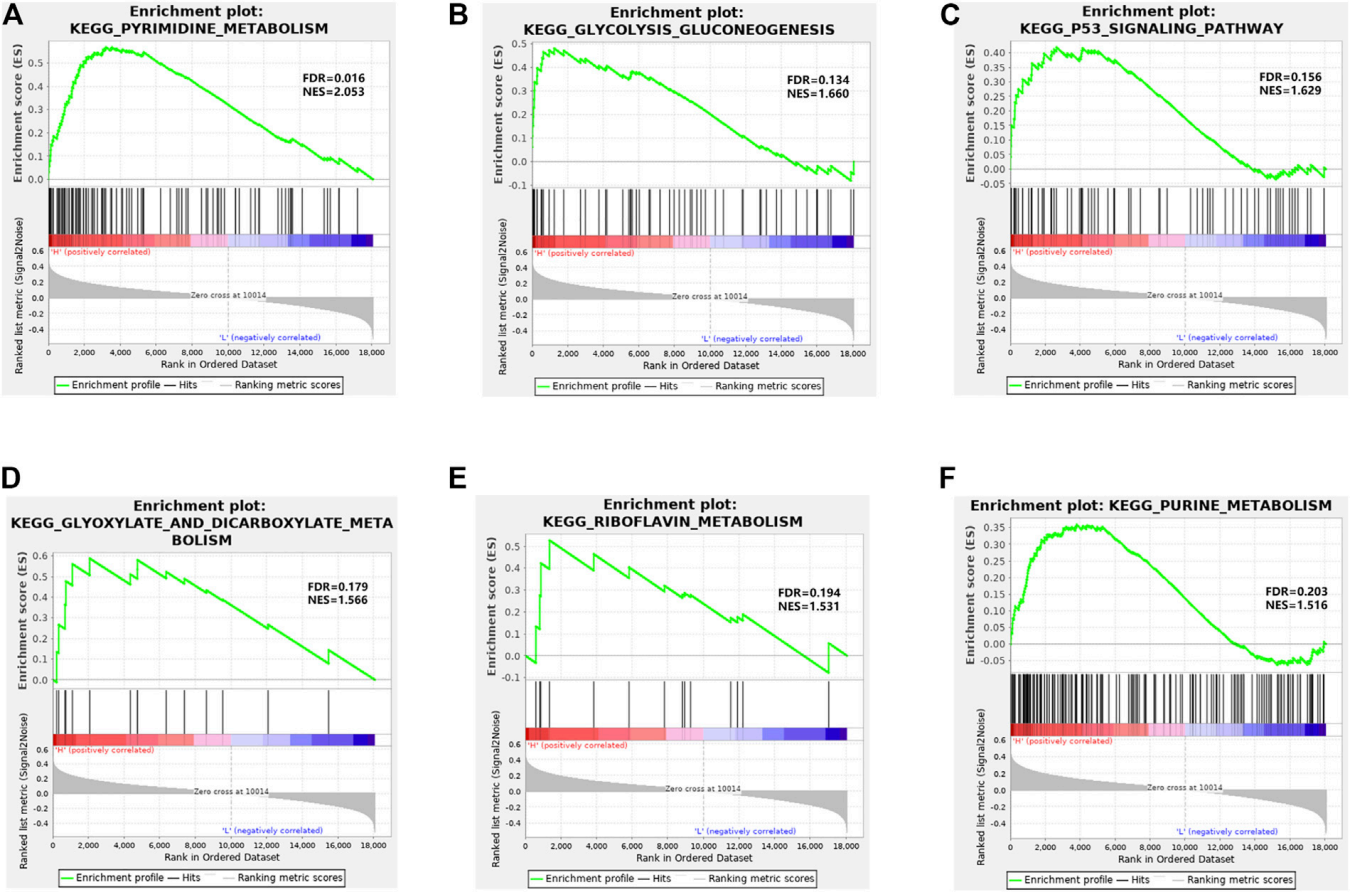
GSEA enrichment analysis. **(A–F)** Enrichment of high- and low-risk groups in pyrimidine metabolism, glycolysis gluconeogenesis, P53 signaling pathway, glyoxylate and dicarboxylate metabolism, riboflavin metabolism, and purine metabolism, respectively.

Based on KEGG pathway enrichment analysis, the high- and low-risk groups displayed significant differences in pathways like pyrimidine metabolism, glycolysis gluconeogenesis, P53 signaling pathway, glyoxylate and dicarboxylate metabolism, riboflavin metabolism, and purine metabolism (Figures 4A–F). These pathways were mostly relevant to signaling pathways like cell carbohydrate metabolism pathway, lipid metabolism pathway, and P53 signaling pathway relevant to cell cycle, apoptosis, and aging.

### Analysis of Tumor Mutation Bearing and TP53 Mutation

**FIGURE 5:**
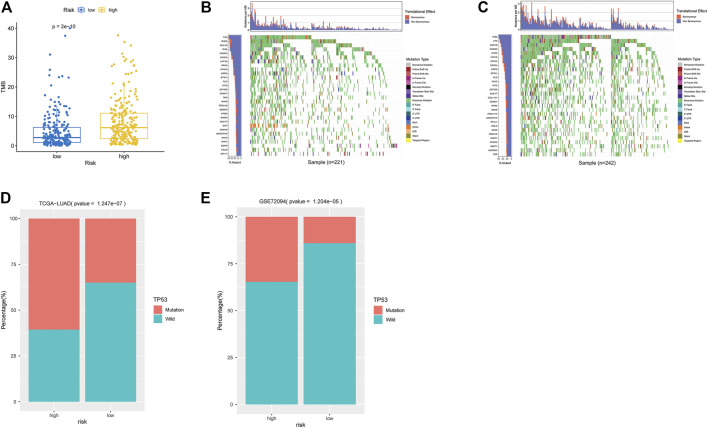
Analysis of TMB and mutation genes. **(A)** Box plot of TMB differences in high- and low-risk groups in TCGA-LUAD dataset. Blue: low-risk group. Yellow: high-risk group. **(B)** Waterfall plot of top30 genes in low-risk group in TCGA-LUAD. X-axis: samples; y-axis: top 30 genes. Different colors of modules represent different mutation types. **(C)** Waterfall plot of top 30 genes in the high-risk group in TCGA-LUAD. **(D)** Histogram of TP53 mutation in high- and low-risk groups in TCGA-LUAD. X-axis: TP53-mutation and TP53-wild in two groups. Y-axis: sample number. **(E)** Histogram of TP53 mutation in two groups of GSE72094 validation set.

As indicated by Wilcoxon test, high-risk groups exhibited significantly higher TMB (Figure 5A). Further analysis on gene mutation revealed differences in the top30 mutation genes in the two groups (Figures 5B,C). GSEA showed that high- and low-risk groups had differences in the P53 signaling pathway. Combining clinical data and SNV data in TCGA-LUAD and GSE72094 datasets, we acquired mutation of TP53 genes in the two groups in two datasets. Chi-square test indicated that TP53 mutation frequency in the high-risk group was evidently higher than that in the low-risk group in two datasets (*p* < 0.001, Table 1 and Figures 5D,E.

**TABLE 1:** TP53 frequency status in high and low risk groups in TCGA-LUAD and GSE72094 datasets.

Gene	Dataset	low Risk Ratio	high Risk Ratio	P value	FDR
TP53	TCGA-LUAD	0.35021097	0.606557377	3.12E-08	1.25E-07
	GSE72094	0.140703518	0.346733668	3.01E-06	1.20E-05

### Differential Expression Analysis of Immune Infiltration

**FIGURE 6:**
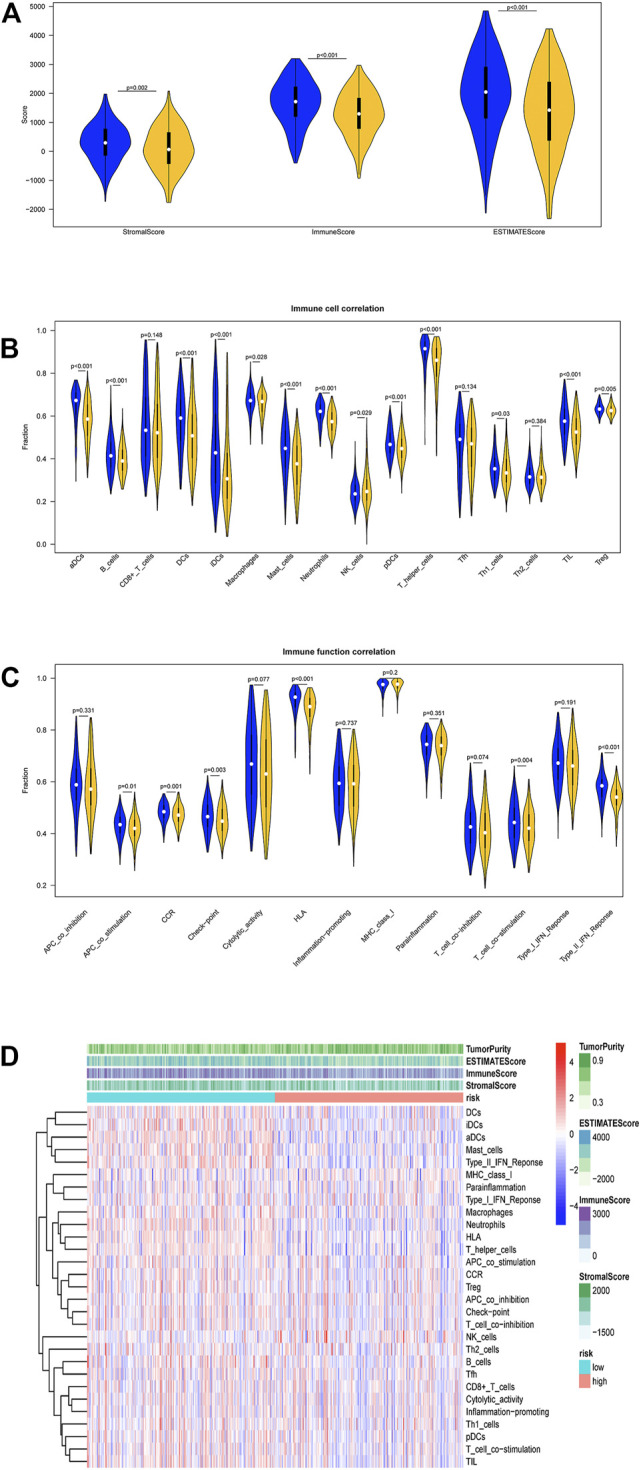
Analysis of differences in immune infiltration in two groups in TCGA-LUAD dataset. **(A)** Differential expression analysis on stromal score, immune score, and ESTIMATE score in the high- and low-risk groups. Blue: low-risk group, red: high-risk group. **(B)** Enrichment levels of 29 immune-related cells and types in two groups. Tumor purity, stromal score, immune score, and ESTIMATE score of each patient in two groups. **(C)** Analysis of differences in immune cell infiltration levels in two groups. Blue: low-risk group, red: high-risk group. **(D)** Analysis of differences in each immune function in two groups. Blue: low-risk group. Red: high-risk group.

R package “estimate” was used to evaluate the infiltration levels of stromal cells, immune cells in TCGA-LUAD samples to acquire stromal score, immune score, and ESTIMATE score. Stromal score, immune score, and ESTIMATE score in the high-risk group were evidently lower than those in the low-risk group (Figure 6A). Subsequently, ssGSEA method was used to analyze the immune activity of LUAD samples. Enrichment levels of 29 types of immune cell sets were acquired. Differences in immune infiltration and activity of these 29 cells in the two groups were also compared. Stromal score, immune score, and ESTIMATE score were decreased with the elevation of risk score, whereas tumor purity was increased. the low-risk group showed higher immune infiltration levels (Figure 6B). In detail, immune cells like T helper cells in the low-risk group had higher infiltration levels (*p* < 0.001, Figure 6C), and most immune function products such as human leukocyte antigen (HLA) had higher expression level (Figure 6D). In summary, the low-risk group showed higher immune activity, which may lead to better prognosis.

## Discussion

With the development of scientific research, it has been found that researching a direction solely (such as genome, proteome, transcriptome) cannot explain all biomedical problems. From a comprehensive perspective, analyses of interaction between genes, proteins, and molecules also cast light on the pathogenesis of human diseases. The bioinformatics method emerged as required by time. Biomarkers found by this method greatly enhance tumor research efficiency. To date, the establishment of cancer prognostic models has been a mainstream of tumor research. For instance, Zheng et al. (2021) identified 12 prognostic feature genes associated with ferroptosis in low level glioma. Jiang et al. (2019) analyzed the glycolysis gene expression profiles of hepatocellular carcinoma and acquired a prognostic model based on metabolism-associated feature genes. This investigation combined tumor metabolism-associated gene sets and TCGA-LUAD dataset to identify metabolism-associated prognostic markers in LUAD and established a five-gene-based prognostic model. The results of this investigation cast light on the research and development of novel biomarkers of LUAD.

TP53 is a common mutation gene in tumors (Giacomelli et al., 2018). We analyzed TP53 mutation in two groups. The high-risk group showed high TP53 mutation frequency whether in TCGA-LUAD or GSE72094. TP53 mutation is an adverse prognostic factor for advanced non-small-cell lung cancer (NSCLC) (Jiao et al., 2018) and a hallmark event of advanced sporadic colon cancer (Watanabe et al., 2019). Moreover, Haupt et al. (2019) found that high TP53 frequency and P53 network dysregulation trigger low survival rate of male cancer patients in North America. It is worthy to note that GSEA enrichment analysis also showed differences in P53 signaling pathway in the high- and low-risk groups. A study also found important functions that P53 performs in metabolism homeostasis. P53 inhibits aerobic glycolysis and stimulates oxidative phosphorylation via several mechanisms to offset the Warburg effect of cancer (Berkers et al., 2013). Thus, we speculated that P53 signaling pathway was inhibited by high TP53 mutation frequency in the high-risk group. Therefore, the role as an inhibitor that P53 played was hampered leading to poor prognosis of the high-risk group.

Based on GSEA enrichment analysis, the two groups mainly showed differences in pathways like pyrimidine metabolism and glycolysis gluconeogenesis. Enhanced Warburg effect and nucleotide metabolism are considered as markers of cancers (Lu, 2019; Siddiqui and Ceppi, 2020). A reference reported that enhanced Warburg effect glycolysis accelerates lactic acid accumulation to influence the tumor microenvironment (TME) and may damage immune cell functions in the TME (Vaupel et al., 2019). In our five-gene-based risk score model, GAPDH has been reported as a key enzyme during glycolysis (Zhong et al., 2018). In addition, CARM1-mediated GAPDH methylation inhibits glycolysis in liver cancer cells (Zhong et al., 2018). Pyridine is an important component of RNA. Pyridine metabolism disorder triggers life activities disorders like DNA copy and protein translation, which may also indirectly lead to immune response disorder. Thus, we postulated that enhanced glycolysis and pyridine metabolism were factors for patient’s poor prognosis.

We also analyzed the two groups with respect to immune cell infiltration. It was discovered that the low-risk group had higher immune scores and immune activity, among which immune scores of helper T cell, dendritic cells (DCs), HLA, and C-C chemokine receptor (CCR) were significantly higher than other immune cells. HLA is the expression product of major histocompatibility complex (MHC) class I molecules, which enables to present endogenous antigen and activate CD8+T cells. CD8+T cells can identify infected cells or cancer cells and activate B cells to form different antigens to perform body immunity functions (Rock et al., 2016). Helper T cells abound with cell classifications, among which Tfh cells can generate IL-21 and express Bcl6 to help B cells to form corresponding antigens. Treg cells can regulate immune response to maintain immune cell homeostasis (Zhu and Zhu, 2020). DCs are center modulators of the adaptive immune responses and prerequisite for T-cell-mediated cancer immunity (Gardner and Ruffell, 2016). CCL16, a ligand of CCR1, accelerates the anti-cancer impacts of DCs and macrophages (Cappello et al., 2006). In this investigation, the low-risk group showed a favorable prognosis. The possible cause may be that helper T cells and MHC class I activate CD8+T cells in TME and activate B cells to secrete a lot of cytokines along with CCR regulation.

On the above, this investigation used bioinformatics analysis to screen metabolism-associated prognostic markers of LUAD. The markers can predict patient’s prognosis well and shed light on the development of novel prognostic markers for LUAD. However, these results came from pure bioinformatics analysis and lack of experimental validation. A series of molecular, cellular, and animal experiments were planned for the future to clarify the mechanism of feature genes screened in LUAD.

## Data Availability

The datasets presented in this study can be found in online repositories. The names of the repository/repositories and accession number(s) can be found in the article/Supplementary Material.
